# Invasion strategy and abiotic activity triggers for non-native gobiids of the River Rhine

**DOI:** 10.1371/journal.pone.0183769

**Published:** 2017-09-15

**Authors:** Jan Baer, Frank Hartmann, Alexander Brinker

**Affiliations:** 1 Fisheries Research Station Baden-Württemberg, LAZBW, Langenargen, Germany; 2 Regierungspräsidium Karlsruhe, Fisheries Administration, Karlsruhe, Germany; University of Minnesota, UNITED STATES

## Abstract

The 24 hour activity patterns of three non-native gobiids (round goby *Neogobius melanostomus*, Western tubenose goby *Proterorhinus semilunaris* and bighead goby *Ponticola kessleri*) were assessed over 46 consecutive months between 2011 and 2014 from their occurrence in the cooling water intake of a nuclear power plant on the River Rhine, Germany. In total, 117717 gobiids were identified and classified. The occurrence of all three species varied strongly between sampling years, and species-specific activity triggers were identified. The activity of juveniles of all three gobiids species was positively temperature dependent while adult tubenose goby activity appeared to be negatively temperature dependent. Increasing fluvial discharge in the adjoining main river stimulated the activity of juvenile round goby but inhibited activity of adult tubenose goby. Except for adult bighead goby, activity was also structured by time of day, but with no uniform mean. Meteorological factors such as precipitation, air pressure and duration of sunshine hours had little or no influence on gobiid activity. On selected rare occasions, mainly at night, all three species exhibited pulsed swarming behaviour, with thousands of individuals recorded in the intake water. Round goby swarms exhibited both the highest intensity and the largest swarming individuals, suggesting a potential competitive advantage over tubenose and bighead goby. Electric fishing surveys in natural river stretches corroborated this observation. Negative effects on the native fish fauna were apparent only for the bullhead, *Cottus gobio*.

The activity triggers identified offer a unique insight into the invasion mechanisms of these ecosystem-changing non-native gobiids.

## Introduction

The use of water from natural systems as a coolant in thermal power stations leads to a wide range of ecological impacts on aquatic communities in both the intake and outtake stretches. Thermal loading of cooling water interferes directly with physiological processes of the biota, such as enzyme activity, feeding, reproduction, respiration, growth and photosynthesis [1]. Of still greater potential impact, however, are the losses of various life-stages of invertebrates and fishes captured on intake screens or entrained within cooling systems. It is not uncommon for millions of fishes and crustaceans to be impinged on power plant intake screens each year [2] and many more eggs, plankton and larvae of invertebrates and fishes are also killed. Absolute mortality figures at some power plants have been shown to exceed 10^10^ individuals annually [2].

The huge demand for cooling water by large thermal power stations means most are located near large water bodies. Permanent monitoring of the fish fauna at such locations is time, labour, and cost intensive and methods are limited by the often considerable depth of water, strong currents, impaired visibility and other confounding factors. Standard sampling techniques, such as electric fishing, diving or the use of gill nets, all have different disadvantages rendering them mostly ineffective in this context [3]. However, the sheer volume of intake waters in large thermal power plants, like in nuclear power plants, constitutes a significant sampling of fish stocks in the source river and studies of fish occurrence in cooling water intakes have facilitated the development of important time series [4], with which to study general biological dynamics, such as changes in fish community composition. Nevertheless, most of these studies conducted at nuclear power plants are looking for the indirect effect of thermal loading [5–9], while the cost and logistics of studies looking for the direct effects of the removal of fish from the intake water in nuclear power plants makes such projects comparatively rare [4]. However such data might provide an insight into otherwise unknown dynamics and in particular the invasion strategies of various non-native fishes in large rivers [10,11].

The round goby *Neogobius melanostomus* (Pallas, 1814) is one of the world’s most wide-ranging invasive fish, whose rapid expansion and deleterious ecosystem effects are well documented [12]. However, detailed studies of autecological aspects of invasions such as activity triggers or co-occurrence effects during the colonisation of a new habitat are largely missing [13]. The present study takes advantages of a unique data set generated by hourly screening of fish and lamprey species from the cooling water intake of a nuclear power plant on the River Rhine. In the present study, this highly resolved data is used to document the chronology and the intensity of invasions of three non-native gobiids and to correlate their occurrence with abiotic factors. Goby invasions first reached the area of the sampling station in 2007: first came the Western tubenose goby *Proterorhinus semilunaris* (Heckel, 1837), then the bighead goby *Ponticola kessleri* (Günther, 1861) in 2010 and finally the round goby in 2011 [14]. All three species most probably arrived via the Main-Danube Canal, built in 1992 to connect the Danube with the Rhine system [14]. The goal of this study was to gain a reliable picture of the goby invasion front, in order to better understand general invading mechanisms, and to identify abiotic activity triggers.

## Material and methods

### Ethics statement

Approval of our present study by a review board institution or ethics committee was not necessary because all fish were caught under the permission of the local fisheries administration (F. Hartmann) and all needed qualifications for the involved people (fishing licenses) were checked regularly by the local member of the animal protection committee (F. Hartmann). Based on the local fishery law (Landesfischereiverordnung, §2) all non-native fish has to be removed, therefore all gobiids were stunned by a blow on the head and expertly killed immediately by a cardiac stab according to the German Animal Protection Law (§ 4) and the ordinance of slaughter and killing of animals (Tierschlachtverordnung § 13). No living gobiids were used. Live native fish captured in the cooling water were maintained in 50 L plastic holding tanks, the contents of which were released every six hours in a bucket lowered into the main channel of the river. Electrofishing was conducted under a license from the fisheries administration (Regierungspräsidium Karlsruhe).

### Study area

**Fig 1:**
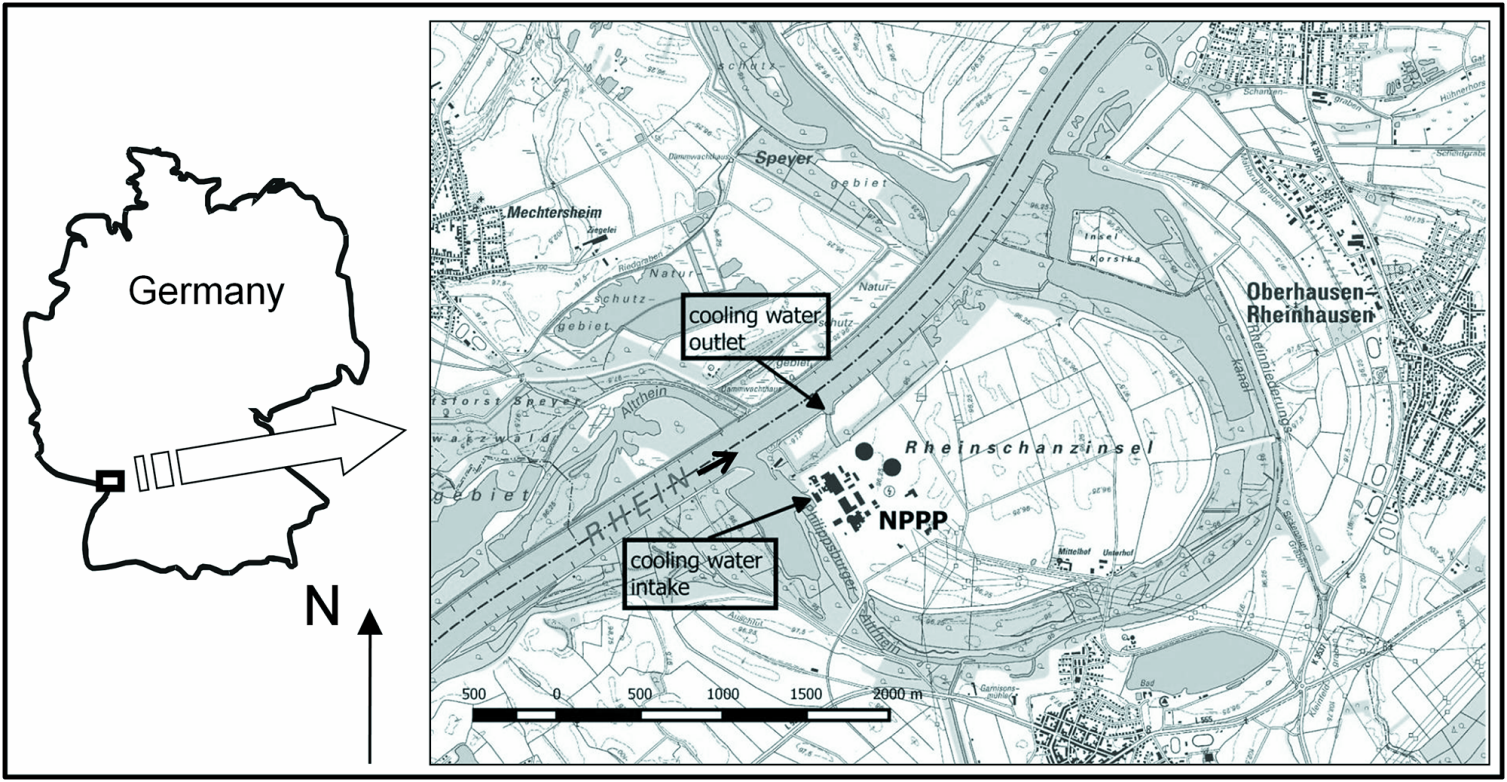
Location of the Philippsburg nuclear power plant (NPPP) in Germany and situation on the River Rhine (here named Rhein, arrow indicating direction of flow).

The Philippsburg nuclear power plant (NPPP) is located in south-western Germany, approximately 30 km north of the city Karlsruhe, on a small island (Rheinschanzinsel), 642.5 km upstream from the mouth of the River Rhine (49°15´N; 08°26´E) (Fig 1). Water from the Rhine has been used for cooling the first (926 Megawatt) unit of NPPP since 1979 and the second (1468 Megawatt) unit since 1984. After the Fukushima nuclear disaster in 2011, the German government decided to abandon nuclear power. NPPP unit 1 ceased operations the same year and its cooling water intake was reduced accordingly. Unit 2 is due to be shut down in 2019 according to German atomic law (Atomgesetz, §7, 1a).

During the study period (01.01.2011–31.10.2014) the cooling water intake of NPPP was between 3 and 96 m³ s-1 (mean: 40.8 ± standard deviation SD 20.9 m³ s^-1^). This water is allowed to be warmed by 8–10°C above the mean river temperature before being discharged. The intake of cooling water happens via an artificial lake connected with the main channel of the Rhine. The lake has a mean wetted with of 250 m and a mean wetted length of 720 m (Fig 1). Water is returned directly to the main river, 500 m downstream of Lake Inlet (Fig 1). The River Rhine adjacent to the mouth of the lake is approximately 220 m wide, with an average depth of 3.75 m and a yearly average discharge of 1265 m³ s^-1^. Despite the location of the cooling intake being on the lake some 300 m from the river, abstraction is considered to take place from the main streaming water body of the Rhine. The intake structure can draw up to 100 m³ s^-1^ of water, comparable to the annual discharge of a medium sized river in this part of Germany, and more than most larger tributaries deliver to the River Rhine. This volume is sufficient to generate an observable flow of water from the main river channel to the intake structure. The intake happens through ten vertical concrete sluices, approximately 5 m high and 3 m wide each. Water is drawn from the full water depth of the artificial lake and transported through different bar racks, at the end of which fishes, crustaceans, plant material and debris are removed by 12 vertical screens with a square mesh-size of 1 mm. Retained material is flushed into a tank, the sampling point.

### Sampling

During the study period, the total body lengths (TL) of all fish longer than 2 cm flushed into the sampling tank were measured to the nearest cm. Individuals of each species and each 1cm length-class were counted. In order to reduce labour-intensive species identification work at times when fish loads were high, fish of less than 2 cm TL were assigned to one generic group called ´fish larvae’. The sampling tank was checked 24 times daily (at the end of each hour), by a team of 20 trained personnel (biologists and supervised students), except during annual NPPP service and maintenance operations when checks were only carried out every second day (15.05.-11.06.2011; 30.05–16.07.2012; 05.05.-04.08.2013; 22.06.-21.08.2014). Hourly sampling was thus achieved on 1308 days.

The NPPP cooling water intake acts as a passive fishing gear [3] and the water includes only migrating or drifting individuals. Thus in the context of this study, the occurrence of an individual in the sampling container is regarded as a proxy for species activity.

Single-pass electric fishing (EFKO 8000, straight DC, 300–600 V, Leutkirch, Germany) was used to determine the catch per unit effort (CPUE, individuals m^-^²) of gobiids at two stretches of the river. Both locations were fished from a boat. These electric fishing surveys focussed mainly on shallow, rocky and sandy areas, the preferred gobiid habitats. These sites were 10 km downstream of the NPPP (near the village of Ketsch, 49°21´N; 08°29´E) and 35 km upstream of the NPPP (near the village of Plittersdorf, 48°53´N; 08°08´E). At the downstream site a 1000–2000 m stretch was fished on eight occasions between 2008 and 2015, while at the upstream site, fishing was carried out on a 100 to 3330 m section six times over the same period.

### Data treatment

Based on a literature review, all sampled gobiids longer than 2cm TL were classified as juveniles (≤ 4 cm TL) or potentially mature individuals (> 4 cm TL) [12,15–17]. The parameters selected for interrogation with multivariate statistics in the current study are: daily arithmetic means of discharge of the Rhine (m³ s^-1^) near NPPP (Pegel Maxau, Wasser- und Schifffahrtsamt Mannheim); water temperature (°C) of the river 20 km upstream of NPPP in (LUBW); daily duration of sunshine hours; precipitation (ml per day) and daily change in air pressure (hPa) compared to the previous day. The latter three parameters were all measured by a weather station 3 km south of NPPP (DWD). Other values incorporated into the analyses were cooling water intake in m³ s^-1^, time of day (night, day, and nautical twilight according to Glarner 2006 [18]) and study year. The general regression model specified the negative binomial distribution for the response variable following the argumentation of O’Hara and Kotze (2010) [19], because of the high number of zero observations. A powerful Lasso technique [20], well suited for large data sets where collinearity is often a problem, was used to condense the independent variables. To assess the effect strength of significant independent variables in the model formula, Monte Carlo samples were obtained by resampling the observed data. This was done under the assumption that factors are uncorrelated and that their likely values would not be represented by a uniform distribution.

All statistics were run on JMP Pro 13.1.0 (64 bit, SAS Institute).

## Results

During the study period a total of 6654592 fish were counted in the sampling container. 45.1% of them (n = 3001388) were larvae (< 2 cm TL, mostly cyprinids) and 46.6% (n = 3102326) were roach (*Rutilus rutilus*), bream (*Abramis brama*), perch (*Perca fluviatilis*) or zander (*Sander lucioperca*) ≥ 2 cm TL. The remainder (8.3%, all ≥ 2 cm TL, n = 550878) belonged to 42 different teleost taxa and three lamprey species. Of 46 identified fish taxa, 33 are considered autochthonous, constituting 84.6% of the natural fish fauna of the Rhine (n = 39), while 13 are allochthonous (Baer et al. 2014). In overall abundance during the whole study period, round goby were ranked in seventh place (n = 90278), bighead goby at thirteenth (n = 14749) and tubenose goby at fourteenth (n = 12690) (Table 1).

**Table 1:** Absolute numbers of the 15 most detected species in the sampling container of the Philippsburg nuclear power plant by study year; the three gobiid species in focus are highlighted in bold.

Species	2011	2012	2013	2014	Total
Roach *(Rutilus rutilus*)	118530	176123	525821	127774	948248
Bream (*Abramis brama*)	51113	93584	538179	227809	910685
Perch (*Perca fluviatilis*)	73693	90751	202005	428251	794700
Zander (*Sander lucioperca*)	169635	33135	50791	195132	448693
Asp (*Leuciscus aspius*)	103932	7417	2366	5791	119506
Bleak (*Alburnus alburnus*)	17771	26151	24216	28515	96653
**Round goby (*Neogobius melanostomus*)**	**1458**	**43033**	**26888**	**18899**	**90278**
Three-spined stickleback (*Gasterosteus aculeatus*)	10190	18505	37750	12207	78652
Common nase (*Chondrostoma nasus*)	28429	2854	3694	5237	40214
Ruffe (*Gymnocephalus cernua*)	6379	4161	11215	271	22026
Sea lamprey (*Petromyzon marinus*)	7290	5972	3596	1752	18610
River lamprey (*Lampetra fluviatilis*)	4405	6158	4065	1244	15872
**Bighead goby (*Ponticola kessleri*)**	**4303**	**2102**	**8047**	**297**	**14749**
**Western tubenose goby (*Proterorhinus semilunaris*)**	**10466**	**1150**	**825**	**249**	**12690**
Common dace (*Leuciscus leuciscus*)	5233	434	1.801	201	7669

Gobiids were recorded in the sampling container almost year round, on 91.5% of study days.

The relative proportions of juvenile gobiids in the sampling tank differed between species: 22.1% of tubenose goby, 28.7% of bighead goby, and 51.1% of round goby were below the 5 cm TL threshold for potential adulthood and were thus regarded as juveniles. Maximum recorded sizes were 20 cm TL for bighead goby, 19 cm TL for round goby and 16 cm TL for tubenose goby.

**Fig 2:**
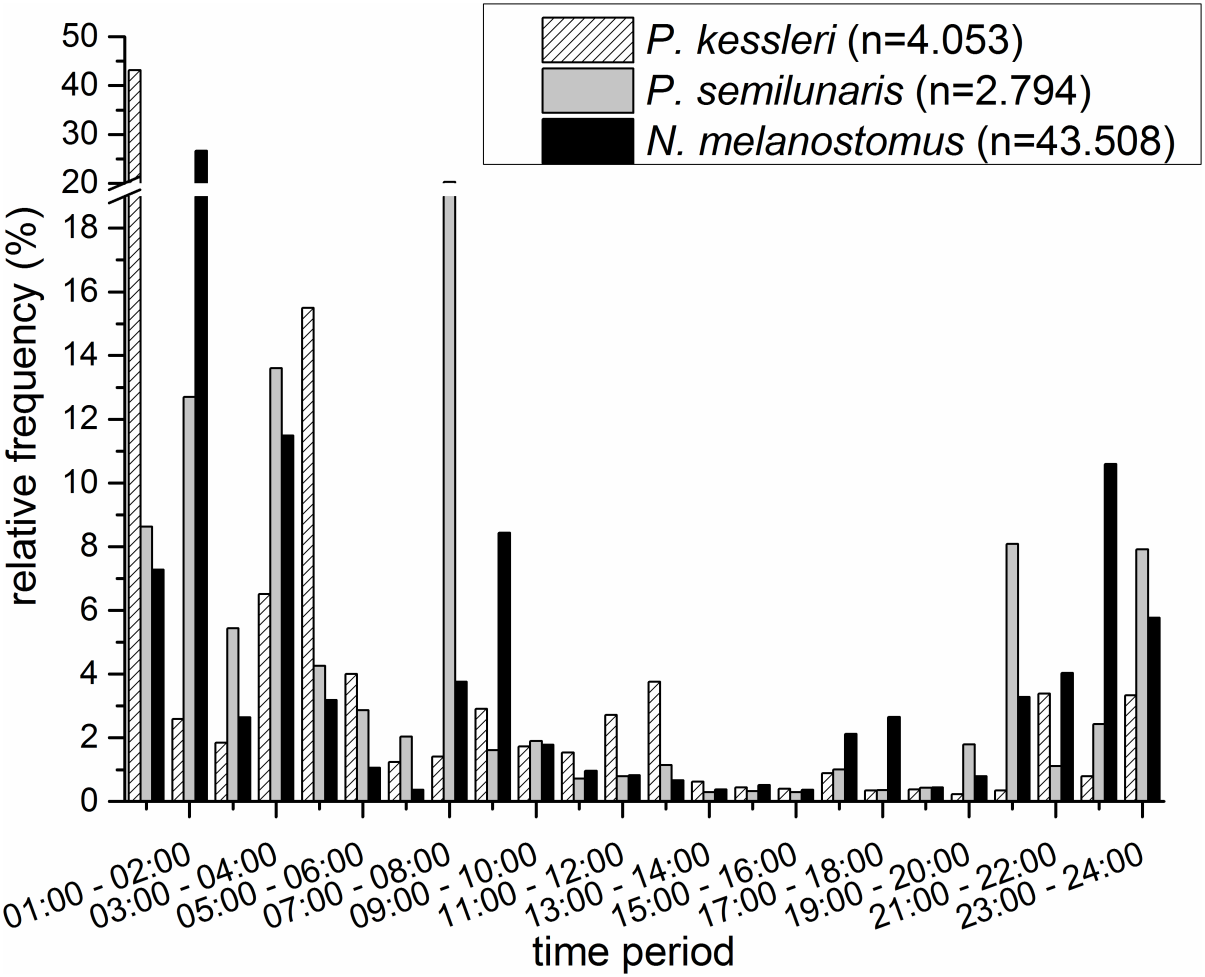
Relative frequencies of juvenile non-native gobiids in an average 24 hour period based on individuals detected in the sampling container during the study period; note the discontinuity of the y-axis, n = sample size.

**Fig 3:**
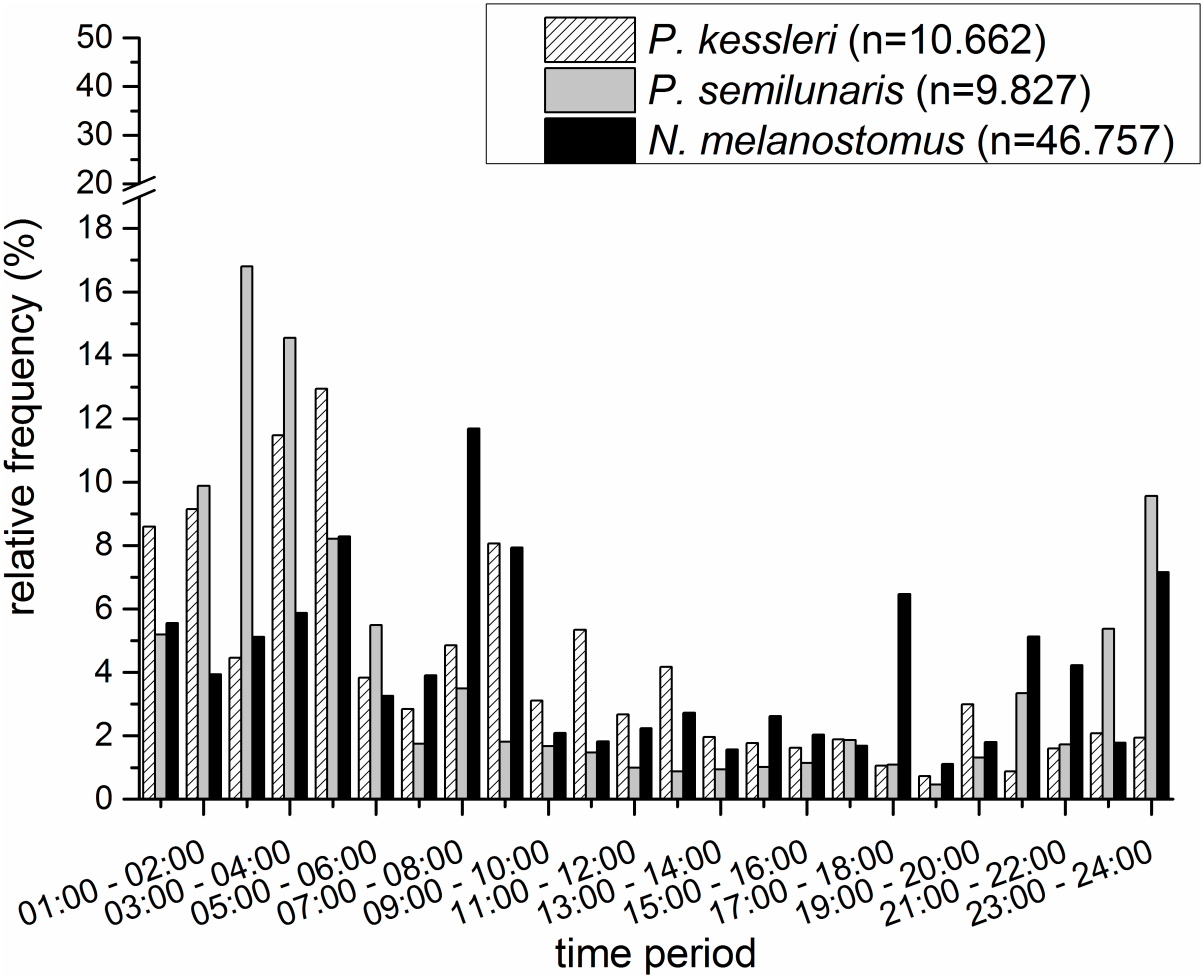
Relative frequencies of adult non-native gobiids in an average 24 hour period based on individuals detected in the sampling container during the study period; note the discontinuity of the y-axis, n = sample size.

The three non-native gobiids occurred at all hours of the day, but the majority were recorded between 9:00 am and 5:00 pm than at other times (Figs 2 and 3). The occurrence of juvenile tubenose goby peaked in the second half of the night (1:00–4:00 am) and around dawn (7:00–8:00 am) (Fig 2); larger individuals were found mainly between 11:00 pm and 5:00 am, but were generally absent at dawn (Fig 3). Nearly 45% of all juvenile bighead goby were detected in the hour just after midnight, with another smaller peak between 4:00 and 5:00 am (Fig 2). Adult bighead gobies were detected around the clock, with small peaks in the latter half of the night and around dawn (Fig 3). The occurrence of juvenile round gobies increased after sunset and peaked one hour after midnight, with a second peak around dawn (Fig 2). Adult round gobies were recorded at all hours, with peaks during the second half of the night and around dawn and dusk (Fig 3).

**Fig 4:**
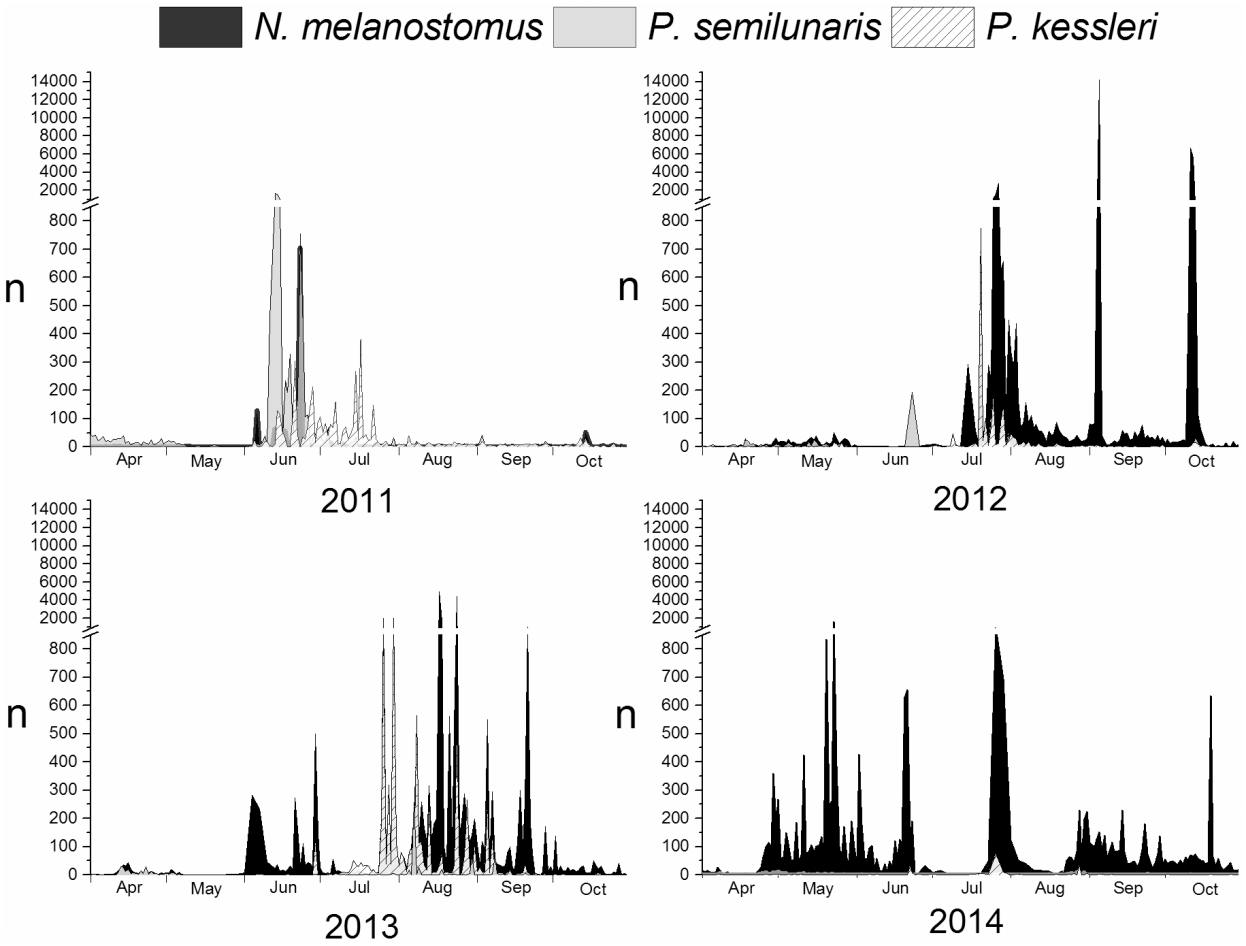
Absolute daily occurrence (n) of gobiid individuals in the sampling container between 1st of April and 31st of October in the four study years; note the discontinuity of the y-axis.

Tubenose gobies were recorded between 1 and 1621 individuals per day (mean ± standard deviation SD: 20.1 ± 516.5) on 631 study days (48.5% of all study days). They appeared mainly during April, May and early June and again in September and October (Fig 4). Bighead goby were found on 598 study days (45.7%), mainly during summer (Fig 4) between 1 and 1968 individuals per day (mean ± SD: 49.2 ± 612.0). Round gobies were recorded on 998 days (76.3%) in densities between 1 and 14193 individuals per day (mean ± SD: 90.4 ± 591.5), mainly during summer or autumn (Fig 4).

**Fig 5:**
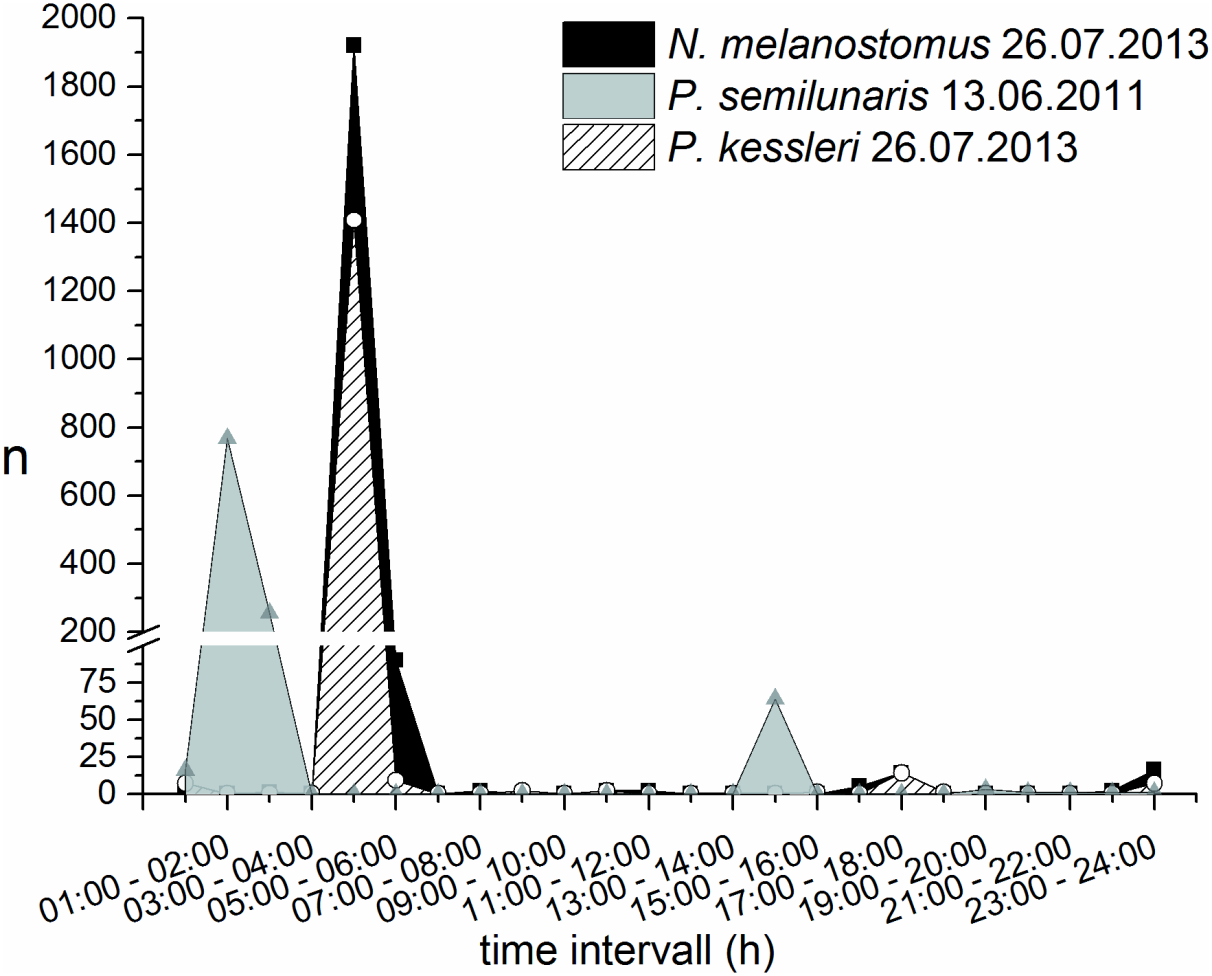
Examples of pulsed swarming behaviour of P. semilunaris on the 13th of June 2011 and of P. kessleri and N. melanostomus on the 26th of July 2013; note the discontinuity of the y-axis.

All three species of invasive goby exhibited pulsed swarming behaviour, with more than 1000 individuals detected within a one to two hour period on a few days in summer (Fig 5) and, in the case of round goby also during autumn. Swarming behaviour was particularly obvious for tubenose goby on three days in 2011, for bighead gobies on two days in 2013 and for round gobies on five days in 2012, five days in 2013 and two days in 2014 (Fig 4). The exceptionally high occurrences recorded at these times were mainly limited to the second half of the night (Fig 5), but on the 18th and 24th of August 2013 more than 1000 round gobies per hour were caught in the morning between 5:00–9:00 am and on one day in October 2012 a similar capture rate was observed around dusk (17:00–18:00 pm). The mean body lengths of individuals recorded during swarming events were 5.1 cm TL ± 0.9 SD for tubenoses and 4.3 cm TL ± 1.1 SD for bighead, with no individuals > 7 cm TL. By contrast, TLs of swarming round gobies ranged up to 14 cm (mean ± SD: 4.4 cm ± 2.5).

Most gobiids detected in the sampling tank during 2011 (Table 1) were tubenose goby (n = 10466), with bighead goby a distant second (n = 4303). Round goby accounting for only 8.9% of all gobiid records for the year 2011 (n = 1458, Table 1). A drastic shift was recorded in 2012 when nearly 93% of all gobiid records referred to round goby (n = 43033, Table 1). In 2013 records of bighead goby peaked at up to 8047 individuals, representing 25% of all gobiids (Table 1). However, round goby remained the most abundant gobiid in 2013 (n = 26888) and by 2014, bighead and tubenose goby had almost disappeared, with round goby making up 97.2% of all gobiid records (n = 18899, Table 1).

**Fig 6:**
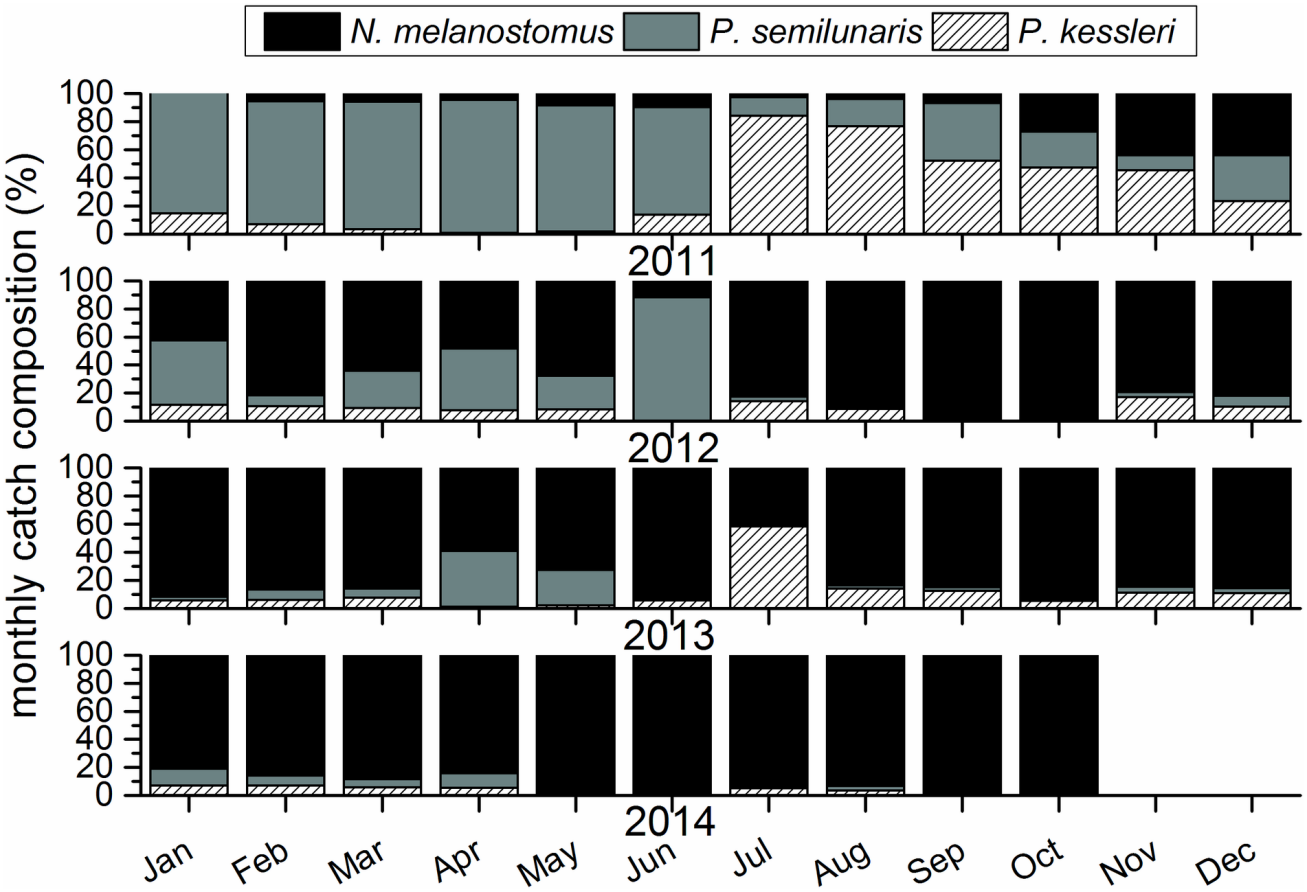
Changes in relative share of the non-native gobiids in the monthly catch during the study period.

In terms of monthly catch composition, tubenose goby dominated the non-native gobiids until July 2011 (Fig 6), at which point a sharp increase of bighead goby was observed resulting in a shift of relative share (Fig 6). However, from autumn 2011, occurrences of round goby also increased and by July 2012 this species began to dominate the catch of non-native gobiids and continued to do so for the remainder of the study period (Fig 6). The exception was summer 2012 (June), when during a phase of generally low gobiid activity, larger numbers of tubenose gobies were found (Figs 4 and 6). A final peak of bighead goby records occurred in July 2013 during nights with over 1000 individuals detected in the sampling tank on some nights (Fig 4), but after that both bighead and tubenose goby records dwindled to almost nothing (Fig 6).

Looking into the model statistics, study year was relevant (*P* < 0.0001) for the consecutive appearance of round, bighead, and tubenose goby (Tables 2 and 3). Water temperature correlated positively (*P* < 0.01) with the occurrence of juveniles of all three species (Table 2) and adults of bighead and round goby (Table 3). In contrast, the occurrence of adult tubenose goby shows a significant (*P* < 0.0001) negative influence of water temperature (Table 3). Fluvial discharge was positively correlated (*P* < 0.0001) with the occurrence of juvenile bighead goby (Table 2) and negatively (*P* < 0.0001) with adult tubenose goby records (Table 3). Time of day appeared to be of minor importance but correlated significantly with the occurrence of juvenile (Table 2) and adult tubenose and round goby (Table 3), though rates differed. The volume of cooling water intake correlated positively with the occurrence of juvenile bighead (*P* < 0.01) and round goby (*P* < 0.0001) (Table 2) and correlated negatively with records of adult bighead goby (*P* < 0.01) (Table 3). The model showed no significant effects of the meteorological factors precipitation, sunshine duration or change in air pressure for tubenose goby, but records of juvenile round goby were negatively affected (*P* < 0.05) by changing air pressure (Table 2), occurrence of adult round gobies increased with precipitation (*P* < 0.01) (Table 3) and the detection of adult bighead goby was negatively influenced by increasing sunshine hours (*P* < 0.0001) (Table 3). The interaction of the two parameters discharge and water temperature had an influence on the detection rate of juvenile round goby (*P* < 0.0001) (Table 2) and adult tubenose goby (*P* < 0.0001) (Table 3). Overall, the model statistics explained 15–20% of observed variation in goby records (Tables 2 and 3).

**Table 2:** The significance and effect strength of different parameters on the rates of detection of juvenile tubenose, bighead and round gobies (whole model: n = 18962, r^2^adjusted = 0.20).

Parameter	tubenose goby		bighead goby		round goby	
	significance/correlation	effect strength*	significance/correlation	effect strength*	significance/correlation	effect strength*
Water temperature	xx/+	0.123	xxx/+	0.756	xxx/+	0.589
Year	xxx/n.a.	0.945	xxx/n.a.	0.355	xxx/n.a.	0.171
Discharge	n.s	0	n.s.	0.052	xxx/+	0.941
Day time	x/n.a.	0.039	xxx/n.a.	0.075	xx/n.a.	0.097
Cooling water intake	n.s.	0	xx/+	0.344	xxx/+	0.260
Precipitation	n.s.	0	n.s.	0.033	n.s.	0.025
Sunshine duration	n.s.	0.010	n.s.	0.050	n.s.	0.059
Change air pressure	n.s.	0.002	n.s.	0	x/-	0.061
Discharge * water temperature	n.s.	0	n.s.	0	xxx/+	n.a.

Model terms: n.s. = not significant; x = P<0.05; xx = P<0.01; xxx = P<0.0001; + = positive correlation; − = negative correlation, n.a. = not applicable.

* dimensionless factor assessing impact of variable in model formula according to Monte Carlo simulations.

**Table 3:** The significance and effect strength of different parameters on the rates of detection of adult tubenose, bighead and round gobies (whole model: n = 18962, r^2^adjusted = 0.15).

Parameter	tubenose goby		bighead goby		round goby	
	significance/correlation	effect strength*	significance/correlation	effect strength*	significance/correlation	effect strength*
Water temperature	xxx/-	0.234	xxx/+	0.572	xxx/+	0.548
Year	xxx/n.a.	0.876	xxx/n.a.	0.563	xxx/n.a.	0.427
Discharge	xxx/-	0.232	n.s.	0.007	n.s.	0.014
Day time	xxx/n.a.	0.161	n.s.	0.035	xx/n.a.	0.068
Cooling water intake	n.s.	0.026	xx/-	0.140	n.s.	0.007
Precipitation	n.s.	0.002	n.s.	0.002	xx/+	0.099
Sunshine duration	n.s.	0.031	xxx/-	0.164	n.s.	0.008
Change air pressure	n.s.	0.003	n.s.	0.001	n.s.	0.010
Discharge * water temperature	xxx/+	n.a.	n.s.	0	n.s.	0

Model terms: n.s. = not significant; x = P<0.05; xx = P<0.01; xxx = P<0.0001; + = positive correlation; − = negative correlation, n.a. = not applicable.

* dimensionless factor assessing impact of variable in model formula according to Monte Carlo simulations.

Electric fishing surveys in the River Rhine in 2008 yielded no gobiid records. The first tubenose gobies were caught upstream of the NPPP (Table 4) in 2009. Bighead gobies began to be recorded in 2010, both up- and downstream of the NPPP, though tubenose goby still dominated the catch (Table 4). In 2011 the first round gobies were found and bighead gobies had increased as a proportion of catch (Table 4). From 2012 onwards round gobies increased enormously and bighead and tubenose gobies nearly disappeared (Table 4). In the recent past (2015) round gobies continued to be found in high densities (0.3–0.5 individuals m^-2^) and tubenose and bighead gobies are comparably rare (Table 4). Another species of goby recently detected approximately 250 km downstream (monkey goby *Neogobius fluviatilis*, Borcherding et al. 2011), was not recorded in this study.

**Table 4:** Relations of the three gobiids recorded in the River Rhine 10km downstream and 35km upstream of NPPP in electric fishing surveys between 2008 and 2015. Figures in brackets indicate number of individuals per m² (CPUE).

	Downstream							
	Sep. 2008	Apr. 2010	Apr. 2011	Apr. 2012	Oct. 2012	May 2014	Oct. 2014	Jul. 2015
Tubenose goby	0% (0)	98.4% (0.0320)	33.3% (0.0040)	0% (0)	0% (0)	0% (0)	0% (0)	0% (0)
Bighead goby	0% (0)	1.6% (0.0005)	55.6% (0.0067)	16.7% (0.0003)	31.7% (0.1088)	0% (0)	0% (0)	0.2% (0.0007)
Round goby	0% (0)	0% (0)	11.1% (0.0013)	83.3% (0.0017)	68.3% (0.2343)	100% (0.2167)	100% (0.0585)	99.8% (0.3333)
	Upstream							
	Sep. 2008	Sep. 2009	Oct. 2011	Oct. 2012	Oct. 2014	Jul. 2015		
Tubenose goby	0% (0)	100% (0.0200)	1.8% (0.0010)	0% (0)	0% (0)	1.4% (0.0075)		
Bighead goby	0% (0)	0% (0)	96.8% (0.0538)	30.3% (0.0464)	0% (0)	1.0% (0.0050)		
Round goby	0% (0)	0% (0)	1.6% (0.0008)	69.7% (0.1069)	100% (0.1045)	97.6% (0.5125)		

## Discussion

Results from the monitoring of the cooling water intake of NPPP agree with those of the electric fishing surveys, and both techniques record the same trends in general abundance of three non-native gobiids, including the rapid decline of tubenose and bighead goby after the appearance of round goby. However, the volume and exceptionally high time resolution of data from the cooling water intake and the interpretation of fish records therein as a proxy for fish activity offers a unique opportunity to investigate potential mechanisms underlying the parallel invasions of tubenose, round and bighead goby.

Borcherding et al. (2013) [10] pointed out the importance of circadian factors in forming the ecological niches of the Rhine’s three invasive gobiids. Information pertaining to the 24 hour activity of these species is scarce, especially outside their native range. Dopazo et al. (2008) [13] reported that the proportion of round goby in nighttime catches was double that recorded during the day. Borcherding et al. (2013) [10] recorded significantly greater densities of round goby in surveys conducted two hours after sunset than in samples taken directly after sunset or during the daytime. Janáč et al. (2013) [21] reported that the drift of larval tubenose and round goby occurred almost completely during hours of darkness and that in both species drift density increased significantly during the first hour after dusk. Detailed knowledge of activity patterns may illuminate invasion dynamics or competition scenarios, and could be critical in assessing potential impacts on native fish species, in creating new strategies to control the invasion or in identifying times when sampling might be most efficient. This study shows that round, tubenose and bighead goby are potentially active all day, but with species specific peaks in activity. Furthermore, juveniles and adults need to be approached separately. The activity patterns of adult bighead goby appear not to be dependent on time of day, but those of tubenose and round goby. Round gobies are much more active around dusk than either tubenose or bighead gobies.

All three gobiid species showed higher activity in the second half of the night than the first, presumably reflecting shared feeding or resting habitats in line with other findings [10] that juveniles of round goby migrate between different feeding habitats during the night.

Following their initial introduction, gobiids are typically sedentary with limited home ranges [22] and only larger individuals show any extensive migration activity [11]. This behaviour is in accordance with the ‘stratified dispersal`theory of gobiid river colonization [12], which progresses by means of short distance drifts by larvae [16] or juveniles [23] and the long-distance active migration described for larger individuals [11,24]. However, the data presented here also suggests a third mode of colonization, a kind of pulsed migration in large swarms. All three species showed this behaviour, whereby thousands individuals, most of them between 2 and 5 cm TL, but occasionally up to 14 cm TL (round gobies) were recorded in samples collected during single hours on single nights in summer (and in the case of round goby also in autumn). In the periods immediately before and after those events, only a handful of tubenose, round or bighead gobies were found despite there being no apparent difference in external conditions. Thus the flooding of river stretches with hundreds or thousands of juvenile and young adults over a short time interval appears to be a triggered movement. Mass migration is common in river fishes [25], and is generally assumed to be a strategy for minimising predation risk [26]. In this instance, all observed swarming events took place during summer or in early autumn (round goby only) and the recorded activity of juveniles of all three species correlated positively with water temperature. In addition on some occasions both round and bighead goby exhibited pulsed swarming at the same time (Fig 4), indicating a shared unknown trigger in both species. While water temperature is known to be an important factor initiating fish migrations in rivers [27], the main trigger of this migration event is unclear. Passive downstream migration is possible but given the mainly regular discharge values recorded during the observed swarm events, active upstream movement cannot be discounted. Further studies are needed to address the nature of pulsed swarming in each species.

The differences in activity recorded for different life stages at particular times and the lack of dependence on external factors suggests that strategies and/or biological drivers controlling activity patterns vary with species. A positive correlation between activity and water temperature up to 24°C was previously shown for round goby in the shallow water areas of the Huron-Erie Lake area [13], and while comparable data for bighead and tubenose goby is not available, similar correlations for those species are assumed. Certainly for bighead goby in the current context, water temperature was the factor with highest impact strength on occurrence. In contrast, activity in the larger tubenose goby increased with declining water temperature. In addition, the activity of adult tubenose gobies showed a strong negative correlation with river discharge. The reason for this relationship is unclear and needs to be addressed by future studies. Possibly migrations of adult tubenose goby into new feeding or spawning habitats during spring and autumn may be triggered by fluctuations in water level or decreasing water temperature. In combination these two environmental factors are known to trigger the migration activity of a variety of cold stenothermic river fish species [27]. In contrast to the activity patterns of tubenose goby, discharge had no influence on adult bighead or round goby. Thus the activity patterns of adult individuals appear to have species-specific biological triggers. Time of day did not appear to influence the activity of adult bighead goby, while sunshine duration did. In contrast, sunshine duration did not influence the activity of larger round or tubenose goby, but the occurrence of adult round goby was positively correlated with precipitation. As sunshine and precipitation both influence turbidity and visibility bighead and round goby may change their behaviour under certain conditions in order to avoid predation—for example becoming less active or resting up in a sheltered location [27,28]. As a general rule, the behaviour and activity patterns of younger gobiids were more similar: independent of species they showed significantly greater activity with increasing water temperatures and exhibited similar activity preferences relating to time of day. Furthermore, in contrast to adult fish, juveniles seemed not to respond to visibility conditions. This might be due the juvenile habit of migrating in large shoals, which in itself may reduce the risk of predation [26–28]. However, there is some species related difference among young stages: in juvenile round goby, activity was stimulated by variations in river discharge and changes in air pressure, a pattern shared with species such as the European eel *Anguilla anguilla* [29], but not in this case with bighead or tubenose gobies, again indicating that different species respond to different triggers. Nonetheless, taking into account the effect strength of all hydrological and meteorological parameters, water temperature remains the main abiotic driver of activity for all three species, with fluvial discharge having higher importance only for juvenile round goby.

It should be acknowledged that the abiotic factors under consideration leave a significant part of recorded variation in gobiid activity unexplained. This variation is probably partly linked to biological triggers which cannot be addressed by the current setup. It is inevitable that some limitations and biases will result from the sampling technique, but the advantages of sample size and time resolution in this study offer fascinating insight into the activity patters of the three species.

Round goby competes with many native species through resource competition, spawning interference and displacement to sub-optimal habitat [12]. Competition with other invasive gobies seems likely, too, because all invasive gobies exhibit a similar sedentary lifestyle [10]. Although interaction between invasive gobies has not yet been studied in detail, that there is strong inter-specific competition for food [10] and space [30] seems obvious. The present investigation revealed a link between occurrence of the different species and the presence or absence of the others, providing further evidence for interspecific competition. Neither of the first two gobiid species to become established was able to hold place in the face of the third invader. The different swarming behaviour of round gobies, whereby swarms occur more often than for tubenose or bighead gobies (up to six times per year compared to once or twice), at a wider range of times, at seven times higher intensities and with larger individuals, might easily confer a competitive advantage contributing to the dramatic decline in abundance of bighead and tubenose gobies following the invasion of round goby. Round goby may outcompete tubenose and bighead goby by sheer number, size, and overcrowding of available space. In addition, while round goby shares a number of food resources with tubenose and bighead goby, including zooplankton (as juveniles), benthic invertebrates, small fishes and the eggs and larvae of large fishes [10,31], they can also consume mussels when more profitable prey is rare or difficult to capture [32]. In the study area, invasive dreissenids (zebra mussel *Dreissena polymorpha* and quagga mussel *Dreissena rostriformis bugensis*), are building huge stocks [33,34] and provide a potentially important food source for round gobies, thus boosting their competitive advantage and providing a convincing explanation for the observed decline in tubenose and bighead gobies following the arrival of round goby.

While negative impacts on the native fish fauna in the region following the successive goby invasions were considered likely, clear evidence of widespread general declines is lacking in the current data (Table 1). Just one native species shows a clear negative trend, the bottom-dwelling bullhead (*Cottus gobio*). Bullheads were recorded in 2011 in reasonable numbers (n = 57), but following the appearance of non-native gobiids, numbers of bullhead decreased sharply, to four individuals in 2012, none in 2013, and just one in 2014. Electric fishing surveys tell a similar story. Prior to the invasion of the non-native gobiids, bullhead was regularly recorded, but has scarcely featured since the invasion of gobiids. Dubs and Corkum (1996) [35] showed that round gobies in the Great Lakes were more aggressive than native mottled sculpins (*Cottus bairdi*), resulting in the demise of the latter [36]. A similar influence over bullhead in the River Rhine seems likely. In addition, Cammaerts et al. (2012) [37] stated that the competition for space with tubenose goby lead to a decline of the bullhead population in the River Meuse, Belgium. Based on those data, a negative impact from the non-native gobiids on the native bullhead seems likely; however, future studies are needed to shed light on the underlying mechanisms.

To the knowledge of the authors the current study is the first to use a nuclear power plant as a permanent monitoring station at the invasion front of non-native fish species. This project has generated a wealth of knowledge in addition to the presented data, including insights into the migration patterns of different lamprey species and new data about locally endangered fish species including Atlantic salmon (*Salmo salar*) and allis shad (*Alosa alosa*) with which to update regional Red Lists [38]. While the costs and manpower required to acquire this data (more than 20 persons were involved) were significant, the results suggest that the cooling water fish screens of nuclear or other thermal power stations have much to offer studies of large rivers fish communities and the mechanisms stimulating activity, which are otherwise extremely difficult to study.

In conclusion, the data presented reveals different activity patterns for round, bighead and tubenose goby and links them to abiotic factors and the disappearance of one native fish species. This data adds to knowledge of the mechanisms underlying the invasion of non-native gobiids and opens new paths to the study of fish community dynamics in the complex setting of large rivers.
